# Analysis of Potential Gene Doping Preparations for Transgenic DNA in the Context of Sports Drug Testing Programs

**DOI:** 10.3390/ijms242115835

**Published:** 2023-10-31

**Authors:** Nana Naumann, Alina Paßreiter, Andreas Thomas, Oliver Krug, Katja Walpurgis, Mario Thevis

**Affiliations:** 1 Center for Preventive Doping Research, Institute of Biochemistry, German Sport University Cologne, 50933 Cologne, Germanythevis@dshs-koeln.de (M.T.); 2 European Monitoring Center for Emerging Doping Agents (EuMoCEDA), 50933 Cologne, Germany

**Keywords:** gene doping, EPO, transgenic DNA, plasmid, sport, black market

## Abstract

Gene doping has been classified as a prohibited method by the World Anti-Doping Agency (WADA) and the International Olympic Committee (IOC) for over two decades. As gene therapeutic approaches improve and, concomitantly, safety concerns regarding clinical applications decline, apprehensions about their illicit use in elite sports continue to grow. Two products available via Internet-based providers and advertised as *EPO*-gene- and *IGF1*-gene-containing materials were analyzed for the presence of potential gene doping agents using a newly developed analytical approach, allowing for the detection of transgenic DNA corresponding to seven potential targets (EPO, FST, GH1, MSTN (Propeptide), IGF1, VEGFA, and VEGFD). Panel detection was based on a 20-plex polymerase chain reaction (PCR) followed by a single base extension (SBE) reaction and subsequent SBE product analyses via matrix-assisted time-of-flight laser desorption/ionization mass spectrometry (MALDI-TOF MS). Extracts of both products were found to contain transgenic *EPO*-DNA, while transgenic DNA for IGF-1 was not detected. The results were confirmed using SYBR Green qPCR with primer sets directed against *EPO* and *IGF1* cDNA, and the CMV promotor sequence. In this case study, the detection of authentic (whilst low concentrated) transgenes, potentially intended for gene doping practices in readily available products, is reported for the first time.

## 1. Introduction

After the first official discussions in 2001 by the “Gene Therapy Working Group” convened by the Medical Commission of the International Olympic Committee (IOC), gene doping has been explicitly prohibited under the regulations of the World Anti-Doping Agency (WADA) in 2003 [1,2,3,4]. Gene therapeutic in vivo approaches are based on the delivery of nucleic acids or nucleic acid analogues into the human body, e.g., via intravenous or direct tissue (e.g., intramuscular) injections. The transfer of transgenic DNA sequences can be achieved using a variety of strategies, including the use of recombinant viral vector systems (such as adenoviral or adeno-associated viral (AAV) systems) or non-viral vectors (such as bacterial plasmids or minicircle DNAs) that can either be transferred “naked” (often assisted by physical force) or complexed with, e.g., cationic lipids or polymers [5,6,7]. As the production, delivery, and expression techniques refine and improve, an increased translation of gene therapeutic approaches into clinical standard care and their commercialization into the pharmaceutical market can be predicted, making gene doping incidents an ever more probable scenario [4,8,9,10].

In the present investigation, two products obtained from Internet-based providers were analyzed for the presence of potential gene doping agents by means of a newly developed multiplex gene doping testing panel. Two different products (here named EPO-P and IGF1-P), presented as nucleic acid-containing formulations suitable for athletic performance enhancement, were ordered via a webpage. EPO-P was described as an *EPO*-gene-encoding plasmid, whereas IGF1-P should provide a plasmid containing the *IGF1* gene. EPO-P was further described to act as a synthetic gene which, upon transcription, yields functional erythropoietin and provides local and systemic effects. CpG motifs within the plasmid should, in addition, positively stimulate the body’s immune system. The products are claimed to be “safe” with no mutational effects on the human genome. Both products should be injected intramuscularly at a concentration of 0.04–2.85 µg/kg for IGF1-P or 1 µg/kg for EPO-P every 5 days. IGF1-P was claimed to be suitable for application in horses as well.

**Figure 1:**
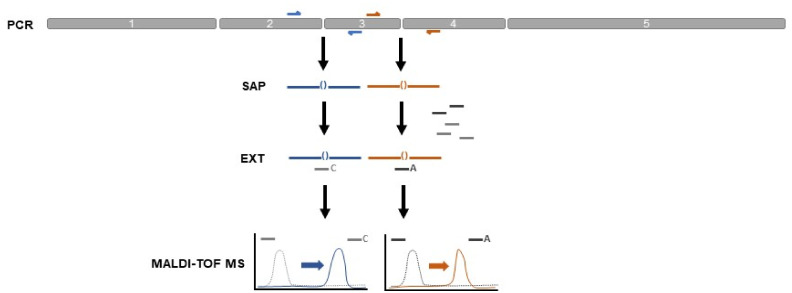
Overview of the gene doping panel detection method. A transgenic DNA sequence, which comprises the sequence information of the five exons of the human *EPO* gene, is exemplarily shown. In a multiplex PCR reaction (PCR), primers directed against the exon–exon junctions 2()3 and 3()4 were used for the generation of transgene-specific amplicons (shown in blue and orange). Upon inactivation of excess dNTPs by shrimp alkaline phosphatase (SAP), extension primers (light and dark grey lines) directed against the exon–exon junction sequences in the PCR amplicons were added to the reaction. In case of the presence of the respective transgenic DNA in a sample, extension primers were single-base-extended (SBE) via ddNTPs in a multiplex extension reaction (EXT). Detection of unextended primers and SBE products was performed using MALDI-TOF MS on a MassARRAY system.

In 2021, WADA approved the first gene doping test protocol for the detection of exon–exon junctions of intronless complementary DNA (cDNA) sequences of the human erythropoietin gene (*EPO*) in whole blood by using quantitative real-time PCR (qPCR) [11,12,13,14]. Other methodological approaches via (nested) end-point polymerase chain reaction (PCR), digital PCR (dPCR), clustered regularly interspaced short palindromic repeats associated with protein-9 (CRISPR-Cas9) system, and Next-Generation Sequencing (NGS) have been developed and tested under research conditions using the same general detection strategy by targeting exon–exon junctions of human cDNA sequences of *EPO* or other gene doping-relevant genes (e.g., *GH1, VEGF-A, FST,* and *IGF-1*) [15,16,17,18,19,20,21,22,23]. Common to most of these approaches, however, is either their limited detection potential concerning the number of targetable exon–exon junctions per reaction (e.g., in the case of qPCR and dPCR) or their costly and technically demanding application (e.g., in the case of NGS). Hence, more comprehensive and high-throughput testing options are desirable. For this reason, a novel high-multiplex gene doping panel was developed in cooperation with Agena Bioscience (San Diego, CA, USA) that enables a multi-target detection method employing matrix-assisted time-of-flight laser desorption/ionization (MALDI-TOF) mass spectrometry (MS) [24]. The new approach is based on a 20-plex PCR amplification step and a subsequent single-base extension (SBE) procedure with a final detection step using MALDI-TOF MS (Figure 1) [25].

Extension primers for SBE product generation are located specifically at exon–exon junctions of relevant transgenic DNA targets. The panel comprises primer assays for seven potential gene doping targets (EPO, FST, GH1, MSTN (Propeptide), IGF1, VEGFA, and VEGFD) with 2–3 detection assays per relevant target transcript in one reaction. For *EPO* transgene detection, the panel includes one detection assay directed against the exon(2)–exon(3) junction (EPO_1 assay) and one detection assay against the exon(3)–exon(4) junction (EPO_2 assay). As several protein-coding transcripts were reported for the human *IGF1* gene, four assays are included in the panel covering the exon(3)–exon(4) junction (IGF1_1_1 and IGF1_1_2 assays), the exon(4)–exon(5) junction (IGF1_2 assay), and exon(4)–exon(6) junction (IGF1_3 assay). A *GAPDH* detection assay is included in the panel as genomic DNA (gDNA) control. Full characterization of the assay is ongoing and will be published elsewhere.

## 2. Case Presentation

### 2.1. Results

#### 2.1.1. Analysis of Nucleic Acid Content in Crude and Extracted Sample Aliquots

Both products were delivered in paper boxes which contained one glass vial of yellowish liquid (~2 mL) each. The products were labelled to contain 1 mg of nucleic acids in 0.9% sodium chloride at a final concentration of 1 mg/mL. However, no nucleic acids were detectable upon DNA purification and analysis with a NanoDrop One system (detection limit of 2 ng/µL according to the manufacturer) and via gel analysis of crude or purified sample aliquots (Table S1). In addition, the samples tested negative for chemically modified nucleotides as well as for steroids and their derivatives.

#### 2.1.2. Identification of Transgenic EPO-DNA by Gene Doping Panel Analysis

**Figure 2:**
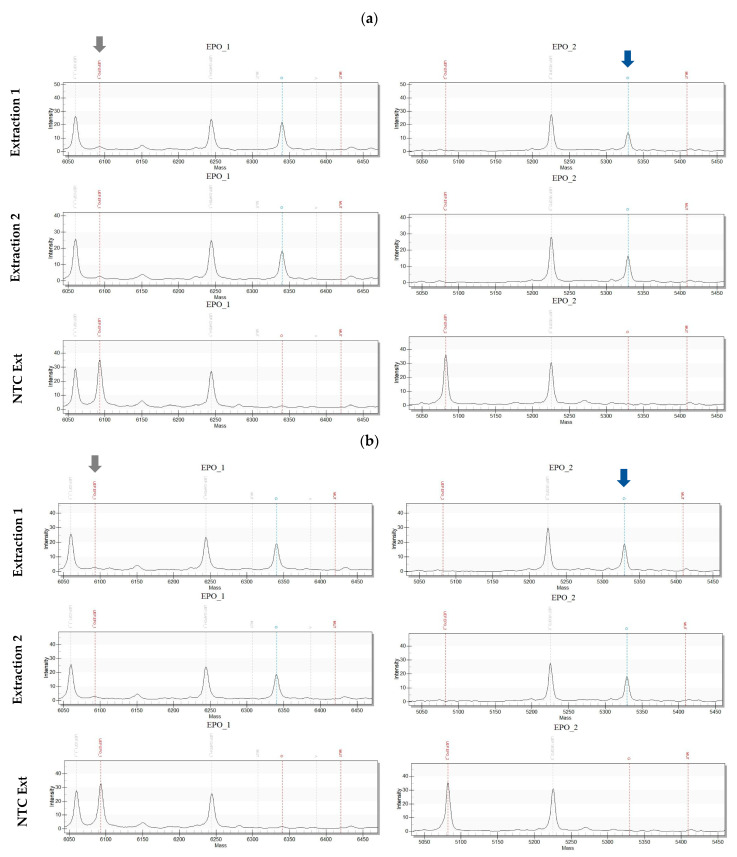
Gene doping panel detection of transgenic human *EPO* via the two *EPO* assays (EPO_1 and EPO_2) in two different sample extracts (extraction 1 and extraction 2) of EPO-P (**a**) and IGF1-P (**b**). Red dotted lines in spectra display expected masses of unextended (**left**, indicated by grey arrow) and extended (**right**, indicated by blue arrow) extension primers, respectively. Blue dotted lines indicate analyte detection at expected mass. NTC Ext = extraction non-template control.

Subsequently, the sample extracts were analyzed with the newly developed gene doping panel. For mass spectrometric transgene identification, 2 μL of each sample extract was added to a multiplex PCR mastermix containing amplification primers for the simultaneous detection of seven potential human gene doping targets, including EPO and IGF1. Amplicons were analyzed following a multiplexed single-base extension (SBE) reaction via MALDI-TOF MS on a MassARRAY System (Agena Bioscience, San Diego, USA) (Figure 1). Panel specificity had been verified by the detection of 160 copies (cps) of human *EPO* and *IGF1* cDNA cloned into the pcDNA3.1(+) vector (Figure S1). Indeed, transgenic *EPO*-DNA was detectable in the EPO-P and IGF1-P extractions via the two exon–exon junction detection assays (EPO_1, EPO_2; Figure 2). IGF1-P had to be concentrated two-fold during extraction to give signals for both *EPO* detection assays (Figure 2b). No *IGF1*-cDNA was detectable in either of the samples (three different exon–exon junctions were tested) nor were any of the other transgenes included in the panel (*FST, MSTN, GH1, VEGFA*, and *VEGFD*). *GAPDH* control assays gave negative results, indicating the samples were not contaminated with genomic DNA (Figure S2).

#### 2.1.3. Verification of Transgenic EPO-DNA Detection via Real-Time PCR

For verification, SYBR Green real-time PCR (qPCR) was performed with primer assays for *EPO*-cDNA detection, as described by Baoutina et al., 2010 (assay 2), and for *IGF1*-cDNA detection, as described by Moser et al., 2014 [13,17]. Human cytomegalovirus (CMV) immediate-early enhancer and promoter regions are ubiquitous vector elements for efficient cDNA expression in mammalian cells and the CMV promoter is one of the most widely used promoters in gene therapeutic vectors [26,27]. Primers directed against the CMV promotor region were thus chosen for additional testing. CMV promoter region primers were designed with the help of a commercial platform. Primer specificity was pretested on the pcDNA3.1(+) control constructs (Figure S3). *EPO*-cDNA and the CMV promoter sequence (pCMV) were also detectable with qPCR in both extractions of EPO-P and IGF1-P (Table 1). The Cq value deviations observed in EPO-P extractions 1 and 2 are most likely due to less efficient construct recovery in the second extraction. Though unspecific products were detectable in NTC extractions of EPO-P and IGF1-P with CMV primer sets upon Cq 38 and 41, respectively, specific detection could be confirmed via fragment size analysis (FSA) and melting curve analysis (unspecific products in NTCs were not of the same size as the expected amplicon; Figure S4). Transgenic *IGF1*-DNA was also not detectable with the qPCR approach.

### 2.2. Materials and Methods

#### 2.2.1. Sample Preparation

Potential nucleic acids were extracted from 100 µL of EPO-P or IGF1-P or 100 µL ultrapure water (as NTC) with the ZYMO DNA Clean & Concentrator™ 25 Kit (Zymo Research, Irvine, CA, USA). Nucleic acids were eluted with 100 µL (EPO-P) or 50 µL (IGF1-P) of ultrapure water. Nucleic acid content was determined by measuring the absorbance at 260 nm (A260) on a NanoDrop One spectrophotometer (ThermoFisher Scientific, Waltham, MA, USA). Additionally, the absorbance at 260 nm (A260) and 280 nm (A280) was measured to test for sample impurities. Sample extracts were stored at −20 °C until further analysis. Sample extractions were performed in a lab area, which had not been used for pcDNA3.1(+) control plasmid preparations up to that point. All surfaces and pipettes were cleaned with DNA AWAY (ThermoFisher Scientific, Waltham, MA, USA) before extraction and fresh filter tip boxes were used. Sample extractions of EPO-P and IGF1-P were performed at different time points.

#### 2.2.2. Gel Electrophoresis

For gel electrophoresis, 2 μL of crude sample or purified extracts or 4 μL of pooled qPCR amplified sample was diluted with water and 2 μL of Hi-Density buffer to a final volume of 10 µL and loaded onto an 8% polyacrylamide gel (Novex™ TBE Gel) along with a GeneRuler Ultra Low Range DNA Ladder (ThermoFisher Scientific, Waltham, MA, USA). Gel electrophoresis was conducted at a 200 V constant voltage for approximately 30 min in TBE Buffer (5× Novex™ TBE Running Buffer, Thermo Fisher Scientific, Waltham, MA, USA) in an XCell SureLock Electrophoresis System (Thermo Fisher Scientific, Waltham, MA, USA) with power supplied by an EPS301 System (Amersham Biosciences, Slough, UK). Subsequently, the gel was washed with HPLC-grade water and stained with SYBR™ Safe (Thermo Fisher Scientific, Waltham, MA, USA). Band visualization was achieved via UV excitation at 302 nm with a Gel DocTM XR+ System (Bio Rad, Hercules, CA, USA).

#### 2.2.3. Gene Doping Panel Analysis

For the panel mastermixes, PCR, SAP, and iPLEX^®^ Pro Reagent Kits of Agena Bioscience (San Diego, CA, USA) were used. Multiplex PCR mastermixes were prepared by mixing 0.3 µL of HPLC-grade water, 0.5 µL of 10 × PCR buffer, 0.4 µL of MgCl_2_, 0.1 µL of dNTP/dUTP mix, 1.0 µL of PCR primers, 0.2 µL of PCR enzyme, and 0.5 µL of UNG enzyme per reaction. A volume of 2 µL of potential nucleic acid extract or ultrapure water (as NTC) was added for a final PCR reaction volume of 5 µL. PCR cycling was performed in a Labcycler Basic System (SensoQuest, Göttingen, Germany) at 30 °C for 10 min and 94 °C for 2 min followed by 45 cycles of 95 °C for 30 s, 56 °C for 30 s, 72 °C for 1 min, and a final elongation step at 72 °C for 5 min. The dephosphorylation of excess dNTPs was achieved by adding 1.53 µL of HPLC-grade water, 0.17 µL of SAP buffer, and 0.3 µL of SAP enzyme per reaction and incubating for 40 min at 37 °C. The SAP enzyme was then inactivated at 85 °C for 5 min. For the iPLEX extension reaction (EXT), 0.62 µL of HPLC-grade water, 0.2 µL of iPLEX Pro Buffer Plus, 0.2 µL of iPLEX Pro Termination Mix (ddNTPs), 0.94 µL of Extend Primer Mix, and 0.04 µL of iPLEX Pro Enzyme were added per reaction. Cycling was performed as no-dwell PCR at 95 °C for 30 s followed by 40 cycles of five repetitive steps of 94 °C for 1 s and 50° C for 1 s. The final extension step was performed at 72 °C for 30 s. Subsequently, 41 µL of HPLC-grade water was added to the EXT products and the reactions were then further processed and analyzed with the help of a MassARRAY Dx Analyzer 4 (MA4) system with a Chip Prep Module Dx (CPM) (Agena Bioscience, San Diego, CA, USA). SBE reaction products were desalted by adding 13 µL of MassARRAY^®^ Clean Resin for ion exchange and were then dispensed by the CPM on a SpectroCHIP^®^ CPM-96 Array (dispense condition: 1200) coated with 3-hydroxypicolinic acid as a crystallizing matrix [28]. The MALDI-TOF MS employed a UV laser (wavelength = 337 nm) and a maximum number of 30 laser shots aiming at the acquisition of 5 (minimum) to 9 (maximum) spectra of acceptable quality. Data analysis was performed with the help of the TYPER v5.0.2 software and via the generation of genotype area reports. All samples were analyzed in duplicate; NTCs were run as controls. PCR reactions and mastermixes were prepared in a UV-cleaned PCR cabinet. All surfaces and pipettes were cleaned with DNA AWAY (ThermoFisher Scientific, Waltham, MA, USA) and fresh filter tip boxes were used. EPO-P and IGF1-P extract analyses were run on different plates at different time points.

#### 2.2.4. Real-Time PCR Analysis

SYBR Green qPCR was performed by adding 2 μL of each sample extract to 10 µL of Quanta bio SYBR GREEN Fast Mix (low Rox; 2×) (Quantabio, Beverly, MA, USA), 0.5 µL of primer mix (250 nM), and 7.5 µL of HPLC-grade water. Cycling was performed in a Mx3000P qPCR System (Agilent Technologies, Santa Clara, CA, USA) at 95 °C for 10 min followed by 45 cycles of 95 °C for 15 s and 60 °C for 40 s. Melt curve analyses were run at 95 °C for 1 min, 55 °C for 30 s, and 95 °C for 30 s. qPCR primers (assay 2) as described in Baoutina et al., 2010 were used for human *EPO*-cDNA detection [13]. For *IGF1*-cDNA detection, primer sequences described as in Moser et al., 2014 were used [17]. CMV promotor primers were designed with the GenScript design tool accessible via: https://www.genscript.com/tools/real-time-pcr-taqman-primer-design-tool accessed on 23 September 2023 (GenScript, New Jersey, NJ, USA). The primer sequences were as follows (5′ -> 3′): GGGCGTGGATAGCGGTTTGA (fw), CATTTGCGTCAATGGGGCGG (rev). The reactions were run in duplicate together with NTC controls. The expected amplicon lengths for the qPCR assay products were 75 bp (hE, EPO), 83 bp (hIG, IGF1), and 135 bp (hC1, pCMV). qPCR reactions were prepared in a UV-cleaned PCR cabinet with fresh filter tip boxes. EPO-P and concentrated IGF1-P extracts were analyzed at different plate runs and time points.

#### 2.2.5. Specificity Analyses

To assess the specificity of the detection of transgenic DNA by the developed gene doping panel, 160 cps of *EPO*- or *IGF1*-cDNA cloned into the pcDNA3.1(+) vector were analyzed in duplicate as described in Section 2.1.2. (GenScript, New Jersey, NJ, USA; ORF clones: OHu20340C (*EPO*; corresponding transcript NM000799), Ohu26817C (*IGF1*; NM000618), and Ohu27428C (*IGF1*; NM0001111283)). NTCs were run as controls. For specificity analysis with EPO (hE), IGF1 (hI), and pCMV (hC1) primer assays, 1500 cps of pcDNA3.1 (+) control constructs (*EPO*: Ohu20340C; IGF1: Ohu26817C) were analyzed using SYBR Green qPCR as described in Section 2.1.3. The reactions were run in duplicate; NTCs were run as controls.

#### 2.2.6. LC-HRMS Analysis for Chemically Modified Nucleotides

Analysis for chemically modified nucleotides by means of liquid chromatography high-resolution mass spectrometry (LC-HR/MS) was performed according to the method of Thomas et al., 2013 [29]. Briefly, 40 µL of both products was fortified with 10 µL of sodium hydroxide (0.5 M) and incubated at 75 °C for 60 min. Afterwards, the samples were neutralized with 5 µL of HCl (1 M), diluted with 100 µL of water, and analyzed using LC-HR/MS. The analysis was performed with a blank sample (water) and a quality control (QC) reference sample containing guanosine-monophosphothioate (GMPS), uridine-monophosphothioate (UMPS), cytosine-monophosphothioate (CMPS), and adensosine-monophosphothioate (AMPS, all four purchased as reference material from BIOLOG Life Science Institute, Bremen, Germany) for comparison. The blank sample (negative control) ensured the absence of signals in the negative samples, while the QC sample (positive control) yielded the expected chromatographic signals at the respective retention times.

#### 2.2.7. Testing on Steroids and Their Derivatives

The products in question were also analyzed for other doping agents, such as anabolic androgenic steroids, via LC-HR/MS according to the method of Krug et al., 2014 [30]. For gas chromatography, 10 µL of dried samples was reconstituted in ethyl acetate or derivatized with a mixture of N-methyl-N-(trimethylsilyl)-trifluoro-acetamide (MSTFA)/ethanethiol and ammonium iodide, respectively. The samples were screened using high-performance liquid chromatography–electrospray ionization tandem mass spectrometry (HPLC-ESI-MS/MS) using an Accela 1250 series HPLC interfaced via electrospray to a Thermo Scientific TSQ Vantage system (Thermo Fisher Scientific, Waltham, MA, USA). For high-resolution mass spectrometry (HR/MS) experiments, a Thermo Exploris was used, and gas chromatography–mass spectrometry (GC-MS) experiments were performed on a Trace 1310 Gas Chromatograph in combination with a TSQ 8000 Evo Triple-Quadrupole Mass Spectrometer (Thermo Fisher Scientific, Waltham, MA, USA).

## 3. Discussion

In two differently labelled products analyzed for the presence of nucleic acid sequences representing gene doping agents, transgenic *EPO*-DNA was detected with a newly developed gene doping panel utilizing high-multiplex MALDI-TOF MS measurements. The findings were verified via qPCR analysis with *EPO*-cDNA-specific primers. Though one of the products was presented with statements suggesting the content of an *IGF1*-containing plasmid, no transgenic *IGF1*-DNA was detectable with the gene doping panel nor with qPCR analysis. The *EPO*-cDNA amount observed in IGF1-P was lower than in EPO-P, and the IGF1-P sample necessitated a two-fold concentration to generate signals for both *EPO* primer assays in the gene doping panel analysis. The gene doping panel does not allow the quantification of analytes, and no absolute quantification qPCR analysis was performed. However, NanoDrop measurements and a gel analysis of (crude) sample extracts demonstrated that the amounts of nucleic acids were much smaller than the labelled concentration of 1 mg/mL. CMV promoter region detection via qPCR analysis in both products indeed indicated the presence of a promoter-driven vector construct instead of short oligonucleotides. Chemically modified nucleotides were not detected. Cq values of the IGF-P sample extracts tested for the CMV promoter sequence were much lower in comparison to the Cq values of the same extracts tested for transgenic *EPO*. *IGF1*-cDNA corresponding to the transcripts NM_000618, NM_001111283, NM_001111284, and NM_001111285 was not detected with the gene doping panel and no other assay of the panel (i.e., *FST, MSTN, GH1, VEGFA,* and *VEGFD*) generated a signal in the conducted screenings. Empty or even different vector constructs might be present in the product. However, the CMV primers used for the qPCR analysis also showed unspecific product generation in NTCs and analysis should be optimized and validated for further investigations. Overall, two products described and sold as *EPO* and *IGF1* plasmid formulations were found to contain small amounts of transgenic *EPO*-DNA. It should be mentioned that the results were confirmed in different test samples of EPO-P and IGF-P at another time point. These products are explicitly classified as gene doping agents according to M3 of WADA’s prohibited list. To our knowledge, this is the first reported finding of a commercialized product that could potentially be used for gene doping purposes. Though the detected amount of *EPO* transgenes is unlikely to result in performance-enhancing effects, the availability of gene doping products readily accessible via Internet-based providers underlines the urgent need for comprehensive testing strategies and their application in anti-doping.

## 4. Conclusions

With the numerous advances in gene therapy clinical trials and the tremendous progress accomplished in the area of new gene therapy-relevant techniques, e.g., CRISPR/Cas, the gene therapy field is moving forward with considerable speed [9,31]. With the approval of the COVID-19 vaccines mRNA-1273 (Elasomeran/Spikevax, Moderna) and BNT162b2 (Tozinameran/Comirnaty, BioNTech) in 2021, the first nucleic acid-based drugs found worldwide use [32]. Thus, the practical application of gene therapeutics in sports becomes an ever more probable scenario and comprehensive testing options for gene doping practices in doping controls are, therefore, highly desirable. Though viral vectors are still the most commonly used gene therapeutic vector systems due to higher transduction efficiencies and more stable expression, plasmids are still used in a significant number of gene therapy clinical trials, and plasmid-based gene therapeutics have already entered the pharmaceutical market. Prominent examples are Neovasculagen (Cambiogenplasmid/PL-VEGF165, Human Stem Cell Institute; approval in Russia, 2011) or the COVID-19 vaccine ZyCoV-D (Zydus Cadila; first approval in India, 2021), the world’s first approved DNA vaccine in humans [9,33,34]. Plasmids are much easier and cheaper to clone, produce on a large scale, and store [5,34], which also makes them an attractive option for black market production. Indeed, transgenic *EPO*-DNA could be detected in two products (marketed as *EPO*- and *IGF1*-gene-containing plasmid formulations) with a newly developed gene doping testing panel. The presented multiplex panel is currently being validated and is planned to complement the existing testing options in sports drug testing in the near future.

## Figures and Tables

**Table 1:** Transgenic *EPO*- and pCMV-DNA detection in EPO-P and IGF1-P by SYBR Green qPCR analysis. Displayed are Cq values of qPCR duplicates of two different sample extractions of EPO-P and IGF1-P, respectively, analyzed with EPO (hE) and pCMV (hC1) primer assays. NTC-Ext = extraction non-template control, nd = not detected.

	Extraction 1	Extraction 2	NTC-Ext
EPO-P			
hE	25.59/25.51	32.20/31.92	nd/nd
hC1	24.59/24.75	30.48/30.48	38.68/44.46
IGF1-P			
hE	28.29/28.22	28.41/28.37	nd/nd
hC1	23.34/22.97	23.14/23.20	41.24/42.89

## Data Availability

The data that support the findings of this study are available from the corresponding author upon reasonable request.

## Supplementary Materials

The following supporting information can be downloaded at: https://www.mdpi.com/article/10.3390/ijms242115835/s1.
