# Supplementary material for: Analysis of Potential Gene Doping Preparations for Transgenic DNA in the Context of Sports Drug Testing Programs
Source: Int J Mol Sci. 2023 Oct 31;24(21):15835. doi: 10.3390/ijms242115835 (PMC10648417; doi:10.3390/ijms242115835)
Supplement: Supplementary file 1 [file ijms-24-15835-s001.zip › ijms-2658577-supplementary.pdf]

Supplementary Information

Sample	Nucleic Acid (ng/μL)	A260/A280	A260/A230
**NTC_Ext**	0.614 ± 0.109	1.149 ± 0.037	0.421 ± 0.054
**EPO_P_1**	-0.083 ± 0.263	2.885 ± 2.909	2.820 ± 2.463
**EPO_P_2**	-0.203 ± 0.079	0.748 ± 0.313	1.928 ± 1.156
**NTC_Ext**	0.157 ± 0.109	0.780 ± 0.679	0.195 ± 0.606
**IGF1_P_1**	-0.524 ± 0.167	1.217 ± 0.413	-0.447 ± 0.215
**IGF1_P_2**	-0.277 ± 0.093	1.243 ± 0.678	-0.207 ± 0.082

Table S1. Analysis of extracted samples by spectrophotometric measurement on a NanoDrop One System.
NTC_Ext = extraction non-template control

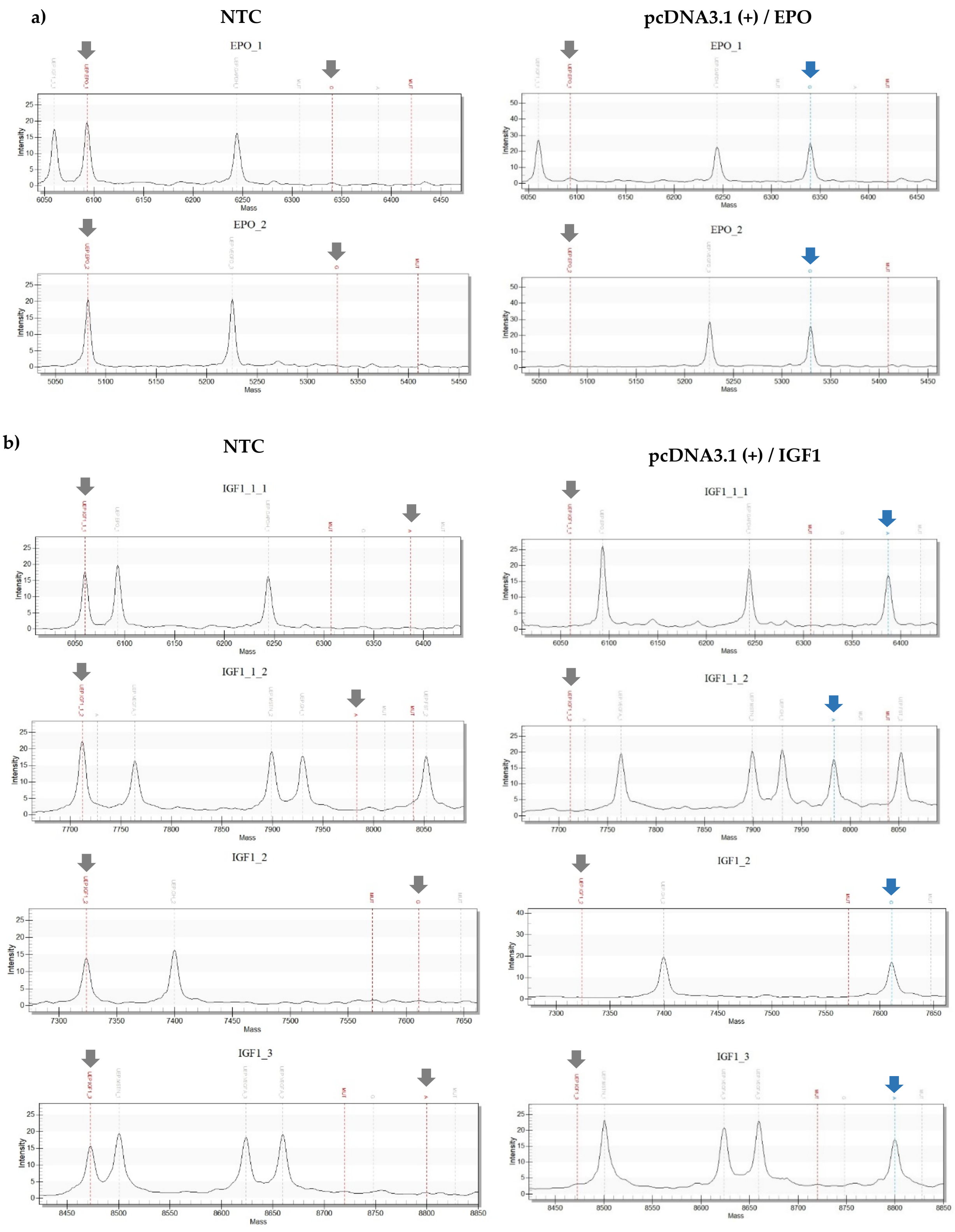

Figure S1. Specificity analysis with the gene doping panel. The panel was tested on 160 copies of *EPO* (a) or *IGF1* (b) cDNA cloned into the pcDNA3.1 (+) vector. Right spectra show non-template controls (NTC) with unextended extension primers. Left spectra show extended primers upon construct detection. Red dotted lines indicated with grey arrows display expected masses of unextended and extended extension primers, respectively. Blue dotted lines and blue arrows indicate analyte detection at expected masses.

a)

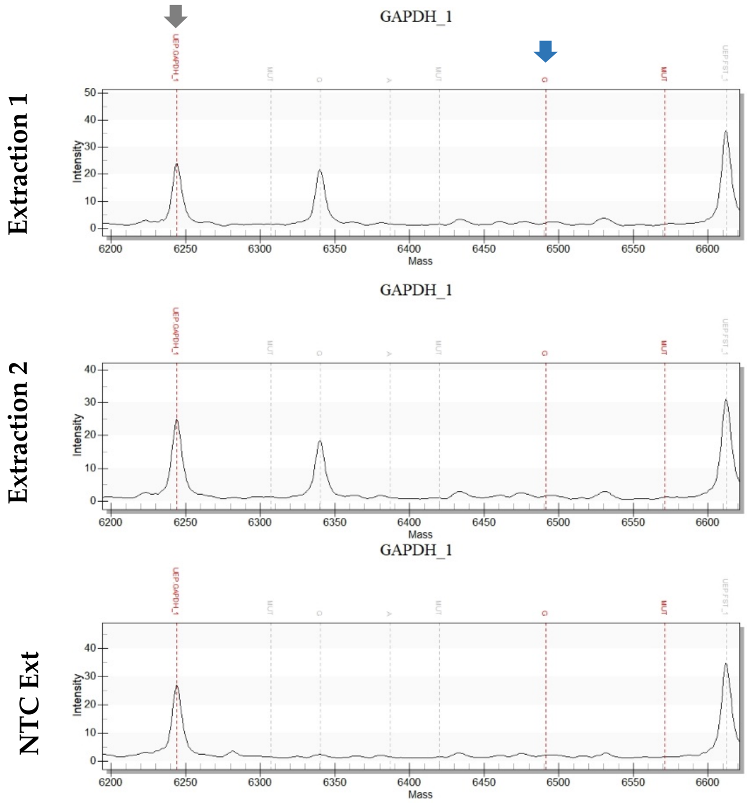

b)

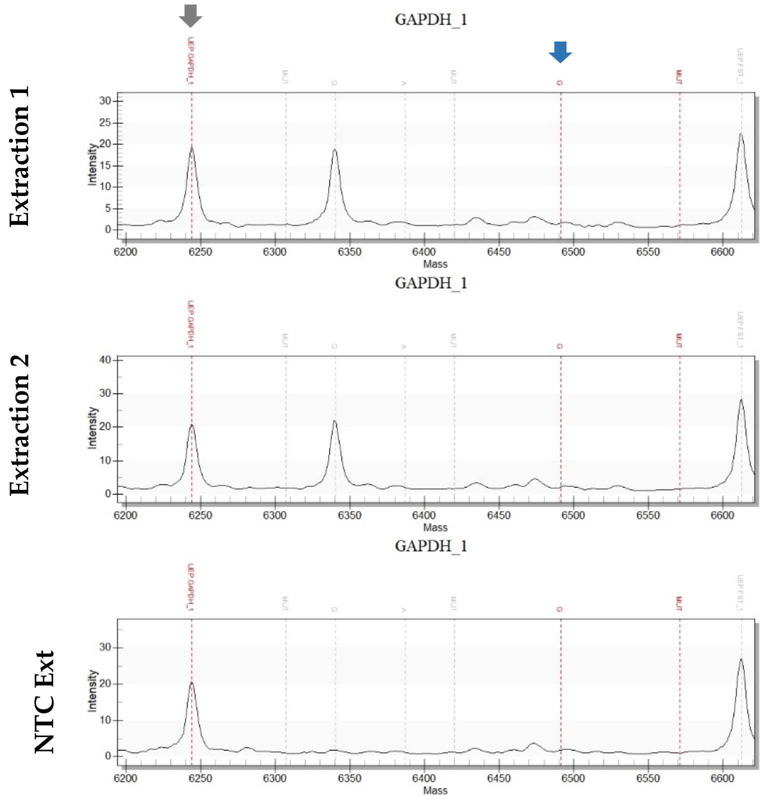

Figure S2. *GAPDH* detection assay results for the two different sample extracts of EPO-P (a) and IGF1-P (b). Red dotted lines on the left and grey arrows indicate expected masses and spectra of unextended GAPDH primers. Expected masses of extended GAPDH primers are indicated with red dotted lines on the right and blue arrows. NTC Ext = extraction non-template control. (Additional peaks seen in the extractions 1 and 2 at an app. mass of 6340 are derived from the extension of EPO_1 primers as transgenic *EPO* was detected in these extracts.)

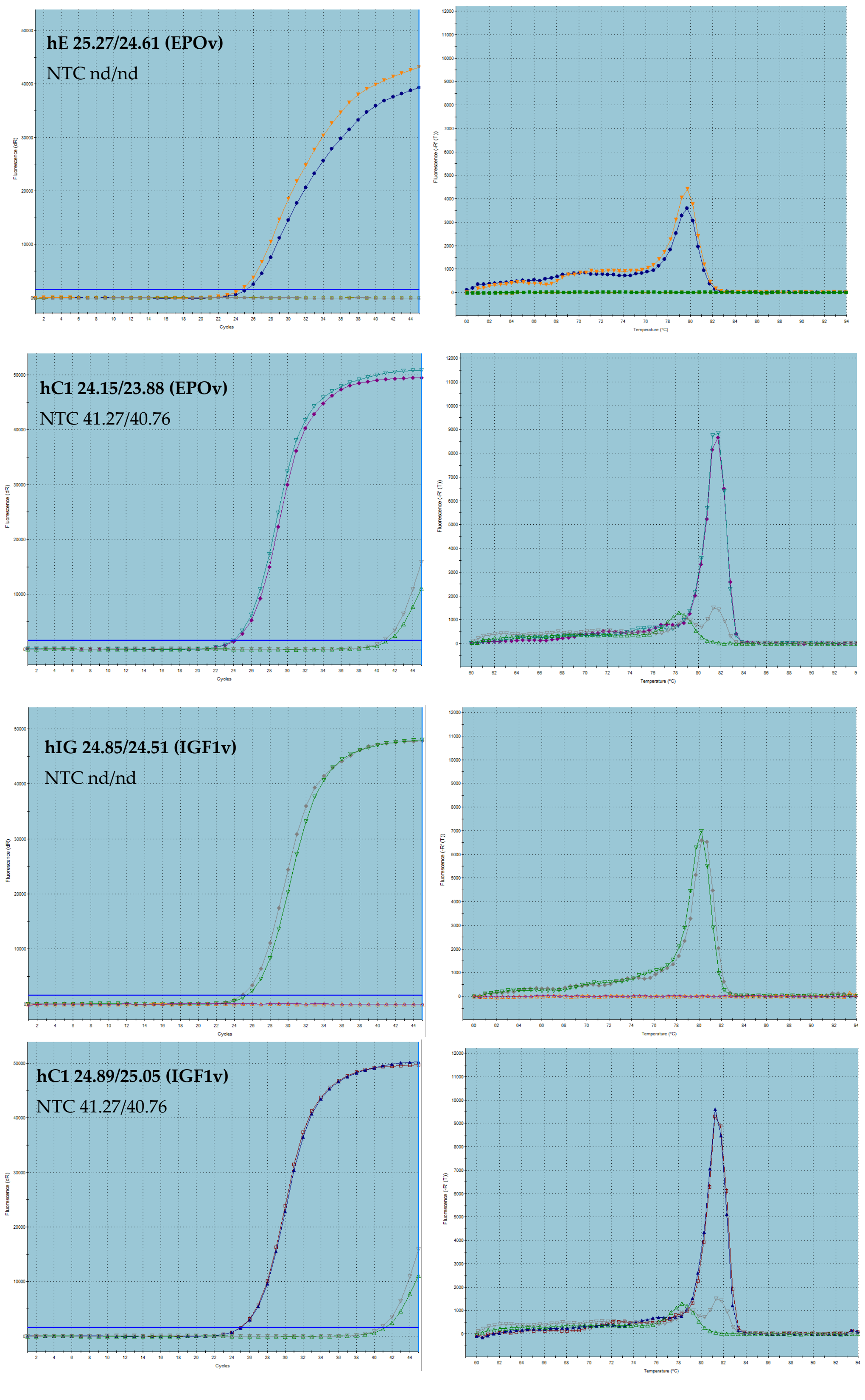

Figure S3. Specificity analysis of primer assays tested by SYBR Green qPCR. EPO (hE), IGF1 (hIG), and pCMV (hC1) primer assays were tested on 1,500 copies of *EPO* (EPOv) or *IGF1* (IGF1v) cDNA cloned into the pcDNA3.1(+) vector. Shown are amplification plots (left) with Cq values and melt curves (right) of duplicate reactions (n = 2). NTC = non-template control, nd = not detected

a)

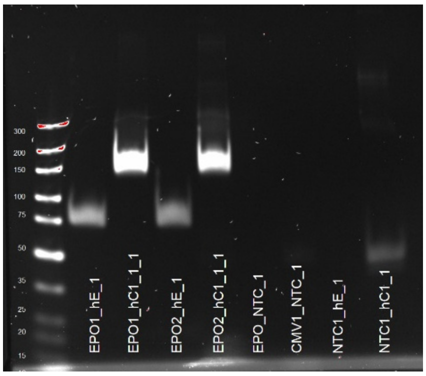

b)

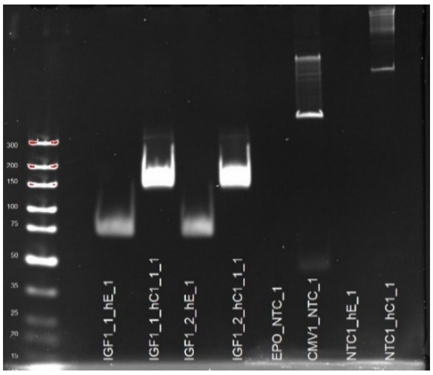

c)

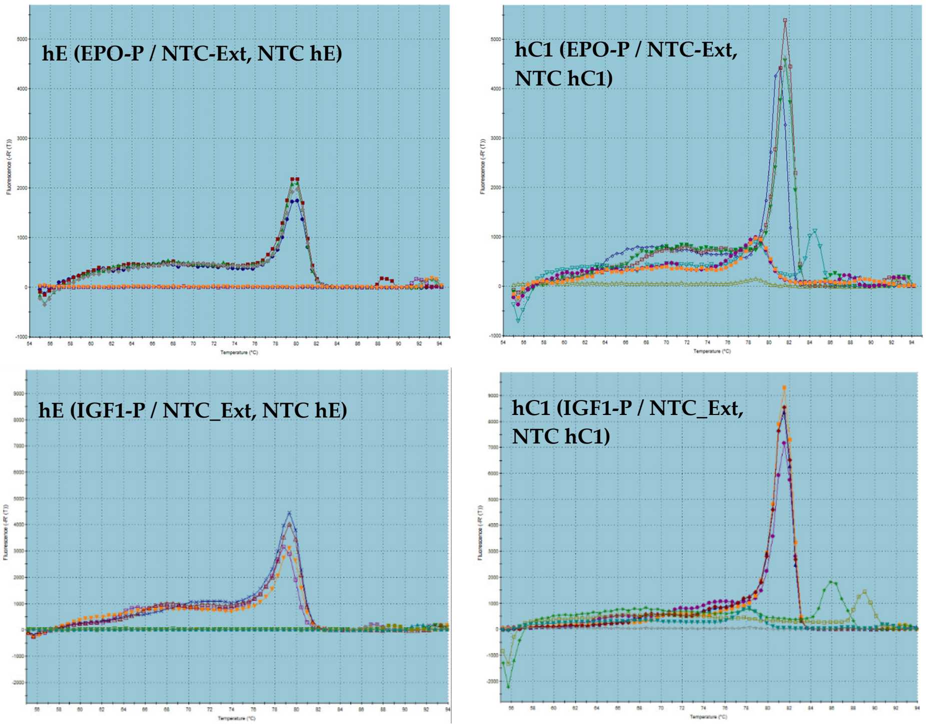

Figure S4. Fragment size analysis (FSA) (a, b) and melt curve analysis (c) of qPCR products of EPO-P and IGF-P. Two extractions (EPO1, EPO2; IGF1_1, IGF1_2) were analyzed with EPO (hE) and CMV (hC1) primer sets in duplicate (n = 2). NTC_1 and NTC_Ext = sample extraction non-template controls; NTC_hE, NTC_hC1 = non-template controls of qPCR.
